# Mechanistic insights into surface contribution towards heat transfer in a nanofluid

**DOI:** 10.1039/d0na00452a

**Published:** 2020-06-10

**Authors:** Ajit Singh, Ramanujam Lenin, Naimat Kalim Bari, Chirodeep Bakli, Chandan Bera

**Affiliations:** Institute of Nano Science and Technology, Habitat Center Phase-X Mohali Punjab – 160062 India chandan@inst.ac.in +91 172 2211074 +91 172 2210075; School of Energy Science and Engineering, Indian Institute of Technology Kharagpur Kharagpur 721302 India chirodeep@iitkgp.ac.in

## Abstract

Nanofluids play a very important role in thermal management and heat exchange processes and for a stable nanofluid, a surfactant is a salient material. There are many contrasting reports on the thermal conductivity of nanofluids and the associated heat transport mechanism in nanofluids. In this article, four different types of nanoparticles are synthesized using citric acid and oleic acid as surfactants, followed by the assessment of their thermal conductivities. For a nanofluid of 3 wt% nanoparticles, coated with citric acid in water 67% reduction in thermal conductivity is observed, and on the other hand a 4% enhancement in thermal conductivity is observed for oleic acid-coated nanoparticles in toluene. This anomaly in the thermal transport behaviour of the nanofluid can be related to the surface properties of nanoparticles and the polarity of the base fluid. Theoretical calculation based on molecular dynamics simulations shows that the reduction in long-range interaction and fluid structuration reduce the thermal conductivity in a polar fluid with a polar surfactant coated nanoparticle.

## Abstract 2

Role of nanoparticle surface in the thermal transport mechanism through nanofluid.

## Graphical abstract

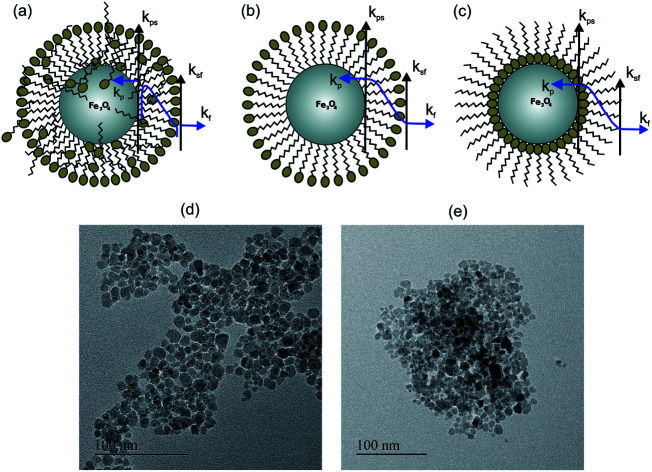

## 1 Introduction

**Fig. 1:**
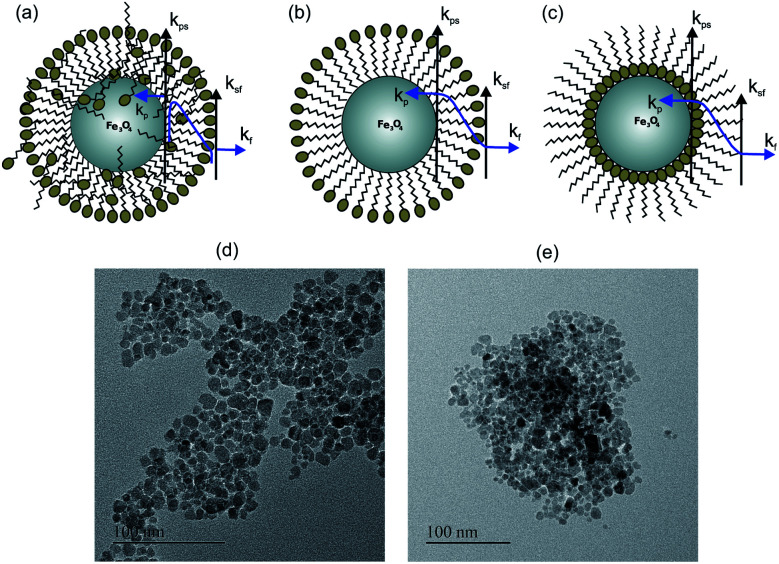
The schematic of the behavior of thermal conductivity at the interface of nanoparticles-surfactant and surfactant-fluid in the base fluids: (a) S1 and S2 for citric acid coated unwashed Fe_3_O_4_ nanoparticles in water, (b) S3 for citric acid coated washed Fe_3_O_4_ nanoparticles in water and (c) S4 for oleic acid coated Fe_3_O_4_ nanoparticles in toluene, (d) TEM image of citric acid coated Fe_3_O_4_ nanoparticles, (e) TEM image of oleic acid coated Fe_3_O_4_ nanoparticles.

Nanofluids have diverse applications in thermal management, thermal insulation and thermal exchange. Recently, they have also gained tremendous attention for biological and clinical applications, where heat transfer plays a critical role. In hyperthermia applications of magnetic nanoparticles, understanding of the heat exchange mechanism between nanoparticles and tissues is important for clinical applications and the heat transfer mechanism also holds the key for drug delivery applications using nanoparticles. Therefore the understanding of thermal conductivity in nanoparticles will lead to the design of many applications and the development of the technology. The thermal conductivity of Fe_3_O_4_ nanofluids has been reported by several groups;^1^ however there is a lack of systematic understanding of the heat transfer mechanism on the thermal conductivity of nanofluids. The remarkable thermal properties of nanofluids predominantly depend on the particle size,^2^ particle morphology,^3^ volume concentration of nanoparticles,^4–6^ and particle surface and temperature.^7^ Different mechanisms are proposed to explain the thermal transport properties of nanofluids such as interfacial resistance,^8^ Brownian motion,^9–12^ liquid layering particle–liquid interface, and nanoparticle clustering.^13–16^ Theoretical models in most of the cases are not aligned with the experimental observation.^17,18^ A few reports of thermal conductivity enhancement in Fe_3_O_4_ and Al_2_O_3_ nanofluids found agreement with effective medium theory,^19,20^ whereas a few reports also highlighted a decrease in thermal conductivity.^21,22^ The thermal properties of Fe_3_O_4_ nanofluids can also be altered with the help of direct and alternating magnetic fields by changing the alignment of the nanoparticles in the fluids.^23,24^ The deterioration of the thermal conductivity can be explained on the basis of the combined effect of interfacial thermal resistance and the presence of a charged surfactant layer on the magnetite nanoparticles (see Fig. 1(a)–(c)). The schematic shows the surfactant coating configuration for different wt% and types of surfactants. Fig. 1(a) and (b) show the citric acid coated (polar particle) and Fig. 1(c) shows the oleic acid coated (apolar particle). The suspension stability of nanofluids is directly affected by the surface charge and surfactant coating.^25,26^ Two types of surface resistance are shown in Fig. 1(a)–(c), where *k*_sf_ is the surface resistance between the surfactant and fluid layer, and *k*_ps_ is the surface resistant between the nanoparticle and surfactant. These two determine the total surface conductance (*G*) of the nanoparticle and have a dominant role in deciding the heat transfer rate through the nanofluid.

The effect of surface charge and surfactant coating on thermal conductivity has not been much explored earlier. In our study, we show the role of surface coating of the nanoparticle on the thermal properties of nanofluids by combination of experimental and theoretical analysis. We have prepared Fe_3_O_4_ nanofluids in water and toluene with citric acid and oleic acid as surfactants respectively to understand the heat transfer mechanism.

## 2 Experimental methods

### 2.1 Materials and methods

For the synthesis of surfactant coated magnetite nanoparticles, iron(iii)chloride hexahydrate (FeCl_3_·6H_2_O), iron(ii)chloride tetrahydrate (FeCl_2_·4H_2_O), citric acid (C_6_H_8_O_7_), and oleic acid (C_18_H_34_O_2_) are purchased from Aldrich Chemicals and ammonium hydroxide (NH_4_OH *i.e.* 30% NH_3_), acetone (C_3_H_6_O), and toluene (C_7_H_8_) are purchased from Merck Chemicals and used as received without any further purification. Distilled water was used throughout the synthesis procedure.

### 2.2 Synthesis of citric acid coated magnetite (S1, S2 and S3)

The synthesis of magnetite nanoparticles involves co-precipitation of the iron chloride salts with an ammonium hydroxide base. The prepared magnetite nanoparticles are then coated with citric acid molecules.^27^ In a typical synthesis, 0.1 M FeCl_3_ and 0.05 M FeCl_2_ were taken in a 500 ml RB flask and heated to 80 °C with constant stirring, and then NH_4_OH solution was added to the reaction mixture. A black precipitate was formed immediately after the addition of the NH_4_OH solution. Then citric acid molecules were added into the reaction mixture, and then the reaction temperature was increased to 95 °C and maintained at that temperature for two hours. The citric acid coated magnetite nanoparticles were settled in the reaction medium. The prepared nanoparticles were separated from the reaction medium using a permanent magnet and then dried and labeled S1 (unwashed sample). By following a similar procedure another batch of the sample was prepared and labeled S2 (unwashed sample). For the preparation of the sample S3, a similar procedure is followed, and in addition to that the product obtained in the previous procedures (S1 and S2) are washed with water using a dialysis process for two days. Then the sample is separated from the aqueous layer and dried in an air oven at 60 °C for 4 h. The dried powder sample S3 (washed sample) was used for different characterization of the sample (sample S3).

### 2.3 Synthesis of oleic acid coated magnetite (S4)

The synthesis of oleic acid coated magnetite nanoparticles involves co-precipitation of iron chlorides using an ammonium hydroxide base in the presence of oleic acid in the reaction medium.^28^ In a typical synthesis, a stoichiometric ratio of 2 : 1 of ferric and ferrous chlorides was taken in a 500 ml round bottom flask and heated to 50 °C. To the preheated solution, the oleic acid surfactant (dissolved in acetone) was added, followed by the addition of the ammonium hydroxide solution (NH_4_OH) to the reaction mixture. A black precipitate was formed immediately after the addition of ammonium hydroxide and then the reaction temperature was increased to 80 °C and continued for 1 hour. After the completion of the coating process the reaction mixture was cooled to room temperature. The product settled in the reaction medium was washed with an acetone–toluene mixture to remove the excess oleic acid on the nanoparticle surface and then washed with water to remove the un-reacted iron chlorides. Finally, nanoparticles were dispersed in toluene and then dried at room temperature to get a dry powder of the oleic acid coated magnetite sample (sample S4). The dried samples were used for further characterization.

The magnetic fluid samples were prepared by dispersing the dried powder samples in an appropriate solvent at different weight percentages. The hydrophilic samples S1, S2 and S3 are dispersed in water whereas the hydrophobic S4 sample was dispersed in toluene at different weight percentages using ultrasonication.

### 2.4 Characterization techniques

The dried samples of S1, S3 and S4 were used for different characterization. The phase purity of the prepared powder samples was analyzed by powder X-ray diffraction using a Bruker D8 Advance X-ray diffractometer having a Cu metal target (Kα radiation, 1.5406 Å). The size and morphology of these three prepared samples were determined by Transmission Electron Microscopy (JOEL JEM-2100) with a working accelerating voltage at 200 kV. The samples for the TEM analysis were prepared in water (S1 and S3) and toluene (S4) and drop casted on a carbon coated copper grid. Fourier Transform Infrared spectra (FTIR) of S1, S3, and S4 were recorded using a Bruker Cary 600 series spectrometer from Agilent Technologies. Dynamic Light Scattering (DLS) and the zeta potential of the samples were studied using a Zetasizer Nano ZSP particle size analyzer and zeta-potential analyzer. For thermal conductivity measurements the surfactant coated nanoparticles are dispersed in appropriate solvents at different weight percentages; the samples S1, S2 and S3 are dispersed in water and the sample S4 is dispersed in toluene. Thermal conductivity measurement of the samples was performed using the well established transient hot-wire measurement technique. We used our home-made hot wire setup for the thermal conductivity measurements of samples. We calibrated the experimental setup using standard fluids such as water and ethylene glycol and compared our results with literature reports.^29^ We observed almost ±1% uncertainty in our experimental results. Heat capacity is measured using a VP-DSC MicroCalorimeter (MicroCal, Malvern Instruments). The sample and the reference cell of a VP-DSC MicroCalorimeter are filled with filtered degassed water and thermal scan is performed in the temperature range of 20–100 °C to obtain a stable baseline. The nanocomposites are extensively dialysed in Milli-Q water (Merk, Germany). The final dialysate buffer is filtered, degassed and filled in both the reference and the sample cell for the blank scan in the 20–100 °C temperature range. Next, the sample cell is filled with respective nanocomposites by injecting with a gastight glass syringe (Hamilton, Reno, Nevada, USA) at 20 °C. The pre-scan thermostat time was set to 10 min to allow for the equilibration of the sample. DSC thermograms were recorded from 20 °C to 100 °C at a scan rate of 1 °C min^−1^. For each set of nanofluids (three different surfactant concentrations) the first higher surfactant concentration was recorded followed by medial and lower. The thermogram of the nanofluid scan was corrected by subtraction of a water-*versus*-water scan. Absolute heat capacity for the same was calculated based on the weight of nanoparticles in the nanofluid.

## 3 Theoretical method

### 3.1 Molecular dynamics simulation

In an effort to mimic nanofluids and decipher the physics of enhancement or attenuation of the thermal transport properties, we use molecular simulations to plot the variation in density and transport coefficients around a particle suspended in a bath of a semi-infinite pool of water. The particle in the simulation domain represents a nanoparticle. The wettability of the particle can be tuned to mimic various types of nanofluids while the surface charge can be used to mimic the properties of the surfactant. The pool of fluid is bounded by walls through which we apply the temperature boundary condition. In a nanofluid, the interaction between the fluid particles and the nanoparticles govern the transport properties. These interactions at the molecular scale are seen to be the interaction of wettability, surface charge, and density distribution. We model our simulations to predict the relationship between these microscopic quantities and how they manifest themselves and continuum behavior. The substrate is modeled using four layers of atoms in the FCC lattice in the 〈100〉 plane. Each unit cell has lateral dimensions of units and the height of water is taken to be 29 units (9.2 nm) from the free surface. Periodic boundary conditions are applied in axial and transverse directions. The number of water molecules in the channel conforms to the bulk density of water at 300 K. Interactions for water molecules are defined using the Simple Point Charge/Extended (SPC/E) model.^30^ The Lennard-Jones (LJ) parameter for the wall atoms and the nanoparticle is taken to be the same as that for water molecules in the SPC/E model. The nanoparticle is formed by a structure in the FCC lattice formed by enclosing all atoms within a radius of units from a central atom.^31^ We test for the size dependence of the nanoparticle on the observed behavior by using the radius of 6 and 8 units also. Surface charge is applied by assigning equal charge to each of the molecules forming the nanoparticle. The heteronuclear interaction with water and wall molecules was further tuned *via* Lennard-Jones (LJ) potential: 
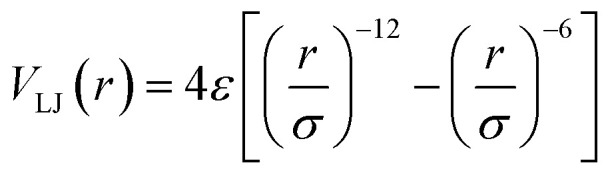
, involving an atomic length scale *σ* and energy scale *ε*. The relevant time-scale for an atomic mass of *m* turns out to be *τ* = *σ*(*m*/*ε*)^1/2^. The wall atoms were thermostated using the Nosé–Hoover thermostat and the fluid molecules dissipate the thermal energy through vibrations of the flexible wall atoms. The long-range electrostatic interactions were obtained using the Particle Ewald Mesh (PME) method. The system was energy minimized and then equilibrated using a leap-frog algorithm for 1000 time units (time steps). Following this, the system was studied using equilibration run for 500 000 time units, integrated using a leap-frog algorithm with a step size of 0.001 units or 500 ns. The time scale was normalized considering *ε* ∼ 48 *k*_B_*T*. The MD simulation platform conformed to an NVT ensemble, consistent with the standard procedure.

### 3.2 Effective medium theory

The effective thermal conductivity, *κ*_nf_, of the nanofluid system is also calculated following effective medium theory including surface conductance.^32^1
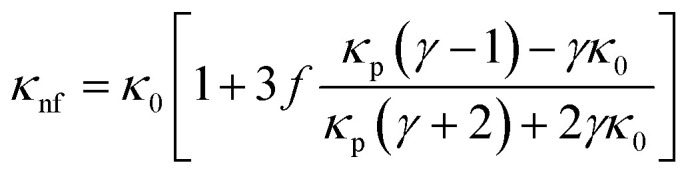
where *κ*_0_ is the base fluid thermal conductivity, *κ*_p_ is the nanoparticle thermal conductivity, *f* is the wt% of nanoparticles, *γ* = *rG*/*κ*_0_, *r* is the nanoparticle diameter, and *G* is the surface conductance. From this model we can estimate the value of *G*, where all other parameters are directly taken from the experiment. The dependence of the nanofluid thermal conductivity on *G* is discussed in the following section.

## 4 Results and discussion

**Fig. 2:**
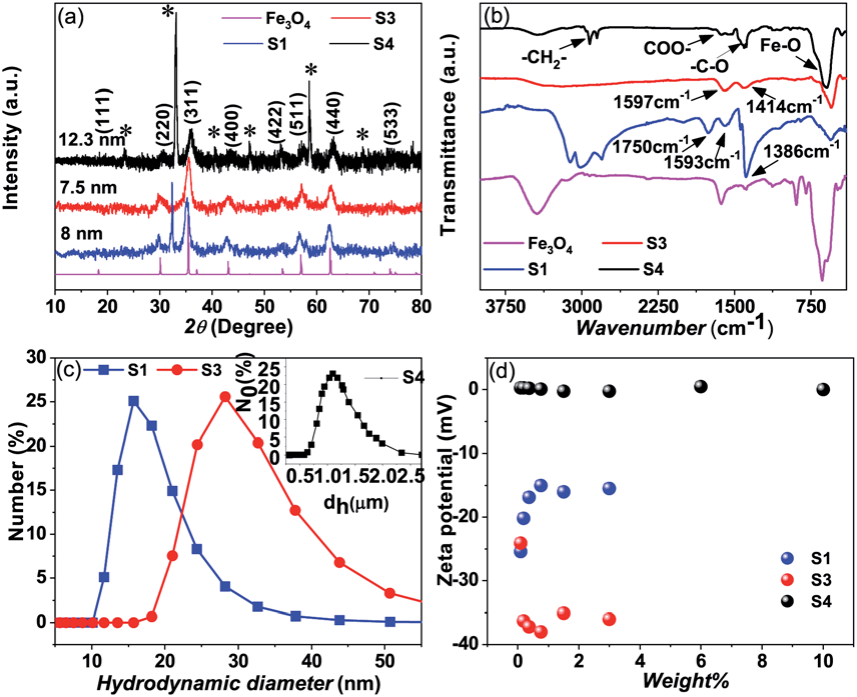
(a) XRD pattern of the prepared magnetite samples S1, S3 and S4, (b) FTIR vibration band spectra for samples S1, S3, S4 and Fe_3_O_4_, (c) DLS of the synthesized samples S1 and S3, and (d) zeta potential measurement for different weight percentages of magnetite nanoparticles of samples S1, S3 and S4.

The XRD pattern of the prepared samples shows the formation of a spinel phase with a lattice parameter of 8.37 Å which is close to the lattice parameter of magnetite (8.40 Å, JCPDS #19-0629) as shown in Fig. 2(a). The average crystallite size calculated from the FWHM of the major peak using the Scherrer equation is 8 ± 1 nm. The added peaks in addition to the spinel peaks are observed in the unwashed sample (S1 and S4). This could be due to the contribution from the excess citric acid and the ammonium hydroxide base, which are denoted by stars in Fig. 2(a) and these additional peaks are not present in the washed sample (S3). The TEM images of the prepared citric and oleic acid coated samples are shown in Fig. 1(d) and (e) respectively. The TEM image shows the formation of approximately spherical particles of a size of around 10 nm. Fig. 2(b) compares the infrared spectra of the washed, unwashed citric acid coated sample and oleic acid coated Fe_3_O_4_ nanoparticles. The band at around 600 cm^−1^ in all coated samples corresponds to the Fe–O stretching frequency and the band at around 1600 cm^−1^ corresponds to –COO^−^ stretching frequency, which indicates that the surfactant molecules are directly attached to the Fe_3_O_4_ nanoparticles through the oxygen atom. Moreover, the band at 1750 cm^−1^ in the sample (S1) corresponds to the free carboxylic acid groups (–COOH), which indicates the presence of free acid molecules on the surface of the nanoparticles in the case of the unwashed sample (S1).^33^

Dynamic Light Scattering (DLS) analysis of sample S1 (un washed) shows almost 17 nm hydrodynamic size with a narrow distribution and the sample S3 (washed) shows a hydrodynamic size of 28 nm with a wider distribution (Fig. 2(c)). Although, the amount of the surfactant in the sample S1 (un washed) is larger than that in the sample S3 (washed), the observed smaller hydrodynamic diameter for sample S1 could be due to the particles that are well separated, possibly individual particles because the large amount of the non-magnetic surfactant layer on the surface of the nanoparticles suppresses the magnetic interaction between the particles and separates them apart by a repelling force of surface charge. But in the case of sample S3, a larger hydrodynamic diameter could be due to the formation of the small clusters/aggregation of the particles in the fluid due to the strong magnetic interaction between the particles with a small amount of the surfactants/charge on the surface.^33^ Similarly for apolar surfactants, there is not much effect of the surfactant on the nanofluid and a large cluster is observed through DLS (see Fig. 2(c)).

The zeta potential measurements of the citric acid coated (S1 and S3) samples and the oleic acid coated sample (S4) with different weight percentages of the samples are given in Fig. 2(d). The washed (S3) citric acid coated sample shows a more negative potential (−35 meV) than the unwashed sample (−14 meV). In the case of the unwashed sample (S1), the zeta potential initially decreases (−24 meV to −14 meV) with increasing concentration and then reaches a constant value with the concentration of the particles in the fluid. Since the sample (S1) is unwashed, increasing the concentration of the particles in the fluid also increases the number of ammonium ions (NH_4_^+^), which partially neutralizes the particle, leading to a decrease in the surface charge. But the observed high negative surface charge in the washed sample (S3) could be due to the negative functional groups (3 COO^−^ groups and one OH^−^ group) present in the citrate ions. Moreover, the sample is washed properly by a dialysis process which removes the excess ammonium (NH_4_^+^) ions and excess citric acid molecules, which means that the particle surface contains only citric acid molecules with high negative charge. The hydrophobic oleic acid coated sample (S4) shows almost zero zeta potential. Although there are larger surfactants on the particle's surface, these particles are dispersed in a non-polar solvent (toluene) which show zero zeta potential.

**Fig. 3:**
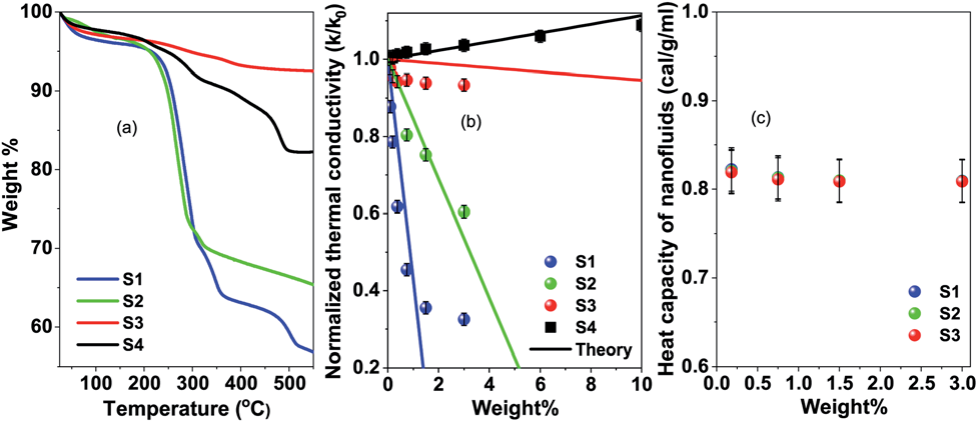
(a) Thermogravimetry analysis of samples S1, S2, S3 and S4, (b) thermal conductivity of samples S1, S2, S3 and S4 with different weight percentages of magnetite nanoparticles, and (c) heat capacity of samples S1, S2 and S3 with the weight percentages of magnetite nanoparticles.

The amount of the surfactants for the washed and unwashed samples is confirmed by thermogravimetry analysis. Fig. 3(a) shows the thermogravimetry analysis of all the citric acid coated samples and oleic acid coated samples. In the case of the citric acid coated sample, the unwashed sample shows a larger amount of surfactant because the excess surfactants in the reaction medium stick to the surface of the nanoparticles as a secondary layer (physically adsorbed) during the drying process. But in the case of the washed sample, the excess surfactants were washed out by water along with the unreacted iron chlorides and ammonium hydroxides and have only less amount of the surfactant (chemically attached primary layer).^33^ The unwashed citric acid coated samples S1 and S2 show 37% and 32% surfactants respectively, whereas the washed sample has only 8% surfactants on its nanoparticle's surface. The oleic acid coated sample shows 18% surfactants on the nanoparticle's surface.

In Fig. 3(b) thermal conductivity measured by the transient hot-wire method is presented for different nanofluids. For the toluene based nanofluid thermal conductivity has linear enhancement with wt% of NPs (black circles in Fig. 3), where the water based nanofluid shows deterioration of thermal conductivity with the wt% of NPs. The measured thermal conductivity of toluene and water is 0.13 W m^−1^ K^−1^ and 0.59 W m^−1^ K^−1^ respectively. Wt% of surfactant on the nanoparticle surface has a dominant role in controlling the thermal transport in the nanofluid system. However, no change in heat capacity at room temperature is observed for these samples as measured by using a VP-DSC MicroCalorimeter (MicroCal, Malvern Instruments) (see Fig. 3(c)). The thermal conductivity, *κ* = *C*_p_*αρ*, linearly depends on heat capacity, *C*_p_, heat diffusivity, *α* and density, *ρ*. It suggests that the thermal diffusivity of the nanofluid system changes drastically in a polar medium. In order to have a better understanding of the heat transfer mechanism and the role of surfactant, molecular dynamics simulations and effective medium theory are integrated with the experiment.

**Fig. 4:**
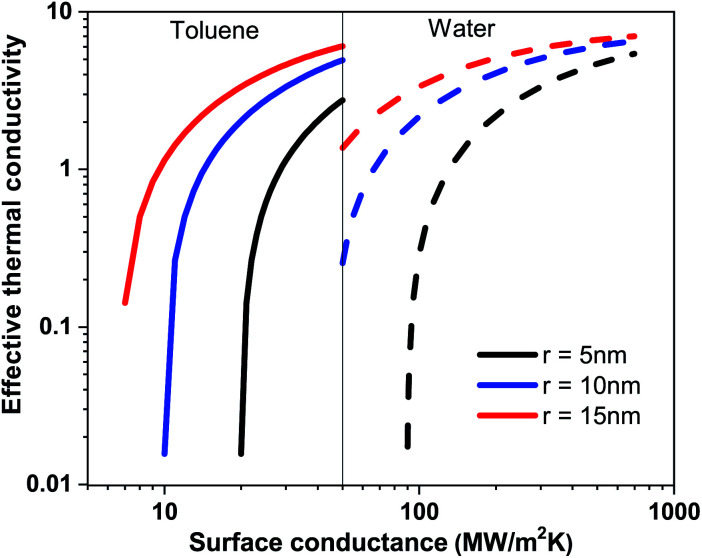
Effective thermal conductivity of the nanofluid based on toluene and water with respect to the surface conductance of nanoparticles at particle radii 5 nm, 10 nm and 15 nm.

In Table 1, the surface conductance *G* of nanoparticle used in eqn (1) to calculate the effective thermal conductivity based on effective medium theory (EMT) is listed. With the increasing surface conductance the effective conductivity of the nanofluid increases. Nanofluid thermal conductivity also strongly depends on the particle size and base fluid (see Fig. 4). In a base fluid with a very low intrinsic thermal conductivity (apolar fluid) nanoparticles with a low surface conductance (>8 MW m^−2^ K^−1^) can increase the effective thermal conductivity. However, for a relatively higher conducting fluid, a higher surface conductance (>80 MW m^−2^ K^−1^) is required for the enhancement of thermal conductivity in nanofluids. As the surface conductance is reduced with the surface coating compared to bare nanoparticles (*G* > 100 MW m^−2^ K^−1^), thermal conductivity is attenuated compared to that of the base fluid. So all the reports with bare nanoparticles in water observed an enhancement of thermal conductivity with nanoparticle wt%. The reduction of thermal conductivity with surfactant coated nanoparticles will be crucial for designing many applications. The EMT shows large changes in thermal conductivity due to the changes in surface conductance for the different wt% of the surfactants on the surface of the nanoparticle. However, the theory is not very consistent at higher wt% of the nanoparticle.

**Table 1:** Measured and calculated characteristic properties of the nanofluids

Sample	Wt% of surfactant	*G* (MW m^−2^ K^−1^)	*κ*_nf_ at 3 wt% (W m^−1^ K^−1^)
S1	37	40	0.20
S2	30	55	0.36
S3	7.5	65	0.56
S4	18	13.4	0.135

**Fig. 5:**
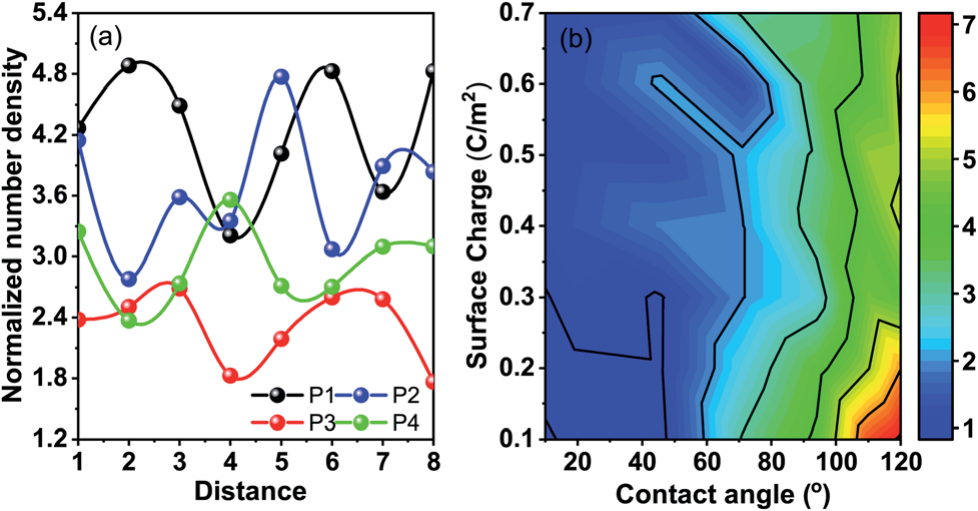
(a) Number density fluctuations of water molecules in the first few molecular layers next to the nanoparticle. The *X*-axis denotes the distance from the nanoparticle normalised in terms of the LJ parameter *Σ*. The *Y*-axis represents the normalized density of water with respect to bulk density. The particles shown are P1: wetting particle with high surface charge; P2: non-wetting particle with high surface charge; P3: wetting particle with low surface charge; and P4: non-wetting particle with low surface charge, while wetting particles induce higher accumulation of water around the nanoparticle, and higher surface charge induces long range ordering. A combination of wetting particles and low surface charge leads to disordered water structuration reducing the thermal conductivity. (b) The colorbar depicts the normalized number density of water with respect to bulk water density. The wettability and the surface charge of the particle are varied to mimic the variation of the chemical properties of the nanoparticle and the surfactant respectively. The wettability is depicted in terms of the static contact angle obtained for a surface with similar chemical properties for a sessile water droplet.

Molecular dynamics (MD) simulations with the LJ potential parameter are used for better understanding of the heat transfer mechanism in the nanofluids. Any transport property of a multi-component system including the thermal conductivity is the reflection of the interfacial free energy distribution.^31^ As one approaches the nanoparticle from the fluid medium the interface gets distorted and this distortion leads to reorganization in the liquid phase leading to number density fluctuations. These number density fluctuations play a key role in deciding the transport properties of the composite system. This can be attested by the fact that heat transfer at the molecular scale can be broken down into four modes, thermal diffusion of nanoparticles, direct collision of nanoparticles, fluid–fluid interactions and fluid–nanoparticle interactions.^30^ The former two are a function of the nanoparticle concentration and other material properties. However, the latter two are function of intermolecular interactions and can be determined from the interfacial energy landscape. In order to understand the physics of energy transport and the dependence of thermal conductivity on nanoparticle–fluid interaction we plot the density distribution of fluid molecules around a nanoparticle. We take into account two distinct factors to understand the interactions in the heterogeneous medium. Firstly, the van der Waals interaction between the nanoparticle and the fluid is taken care by the wettability of the particle. Secondly, the electrostatic interaction between the nanoparticles and the wall is given by the surface charge on the particle which mimics the role of the surfactant in a nanofluid. The wettability of the fluid molecules is represented in terms of the static contact angle that a sessile droplet would form on a flat substrate having the same chemical and physical composition. The surface charge can also be expressed in terms of zeta potential and is taken to be negative without the loss of generality. Fig. 5(a) shows the density profile of water in the first three atomic layers over a distance of 8*σ* for four different combinations of wettability and surface charge. P1 is a particle with wettability corresponding to 8° and a surface charge of 0.6 C m^−2^, P2: 110° and 0.6 C m^−2^, P3: 8° and 0.2 C m^−2^, and P4: 110° and 0.2 C m^−2^. It is observed that for wetting particles there is an enhancement of the number density of water next to the wall as opposed to non-wetting particles where there is a clear depletion of water density next to the wall. The magnitude of the number density peaks depends on the combined influence of wettability and surface charge. A higher surface charge or zeta potential, on the other hand, induces polarization of water molecules leading to a long range order and symmetric arrangement of peaks and valleys in the energy landscape near the particle. Hence one might conclude that the wettability of the particles has a more significant role in deciding the structure of fluid molecules next to the wall while the surface charge decides the ordering of these layers and the correlation between these layers.

In an attempt to comprehend this coupled effect of wettability and surface charge on property variation, we plot the contours for the normalized number density of the first peak next to the particle in Fig. 5(b). The lowest number density peaks are observed for wettable apolar particles. It is observed that with an increase in surface charge the peak density decreases. An increase in surface polarization leads to long range ordering and the peaks are distributed over greater distances giving lower values of individual peaks. Also, the electrostatic interactions override the wettability interactions, reducing the dependence of the peak height on wettability with high surface charge. Apolar particles with low surface charge register the highest peaks next to the particle (followed by the depletion trough) favoured by entropic interactions. This is a result of the coupled interaction of removal of fluid molecules from around the particles and absence of long range ordering. The lowest density peaks are observed for low surface charge and high wettability. The wettability creates a local density peak but the polar water molecules tend to arrange them in a configuration so as to screen the low surface charge of the wall and hence long range ordering is absent. It is for this case that the solid–fluid interaction and the fluid–fluid interaction around the nanoparticle are very small and hence reduce the values of the transport coefficient including the thermal conductivity.

Comparing the same with the experimental results, we can see that the sample S1, the citric acid coated sample, with a low zeta potential registers a decrease in the thermal conductivity value from the base fluid. Interestingly, the enhancement of thermal conductivity is observed in most of the cases where a high degree of fluid structuration and/or long range ordering between the fluid structures enhance the thermal conductivity. The case of wettable particles with low polarity cancels out the effect and causes a decrease in thermal conductivity as can be asserted from the distribution of fluid molecules obtained from molecular dynamics simulations.

## 5 Conclusions

In conclusion, the thermal transport mechanism in nanofluids is investigated by experiment and theoretical analyses. We found that the surfactant plays an important role in the reduction of thermal conductivity in polar (water) nanofluids. Three fold reduction in thermal conductivity is found in water based nanofluids with 3 wt% nanoparticles at 37 wt% of surfactant from 7 wt% of surfactant. However, effective medium theory suggests a very small reduction in surface conductance for 7 wt% surfactant (*G* = 65 MW m^−2^ K^−1^) to 37 wt% surfactant (*G* = 40 MW m^−2^ K^−1^). MD simulations suggest that solid–fluid and fluid–fluid interactions near the nanoparticle reduce the long range interactions and fluid structuration, and hence the thermal transport is also reduced in polar medium with a polar surfactant, hence attesting the experimental observations. In most of the biological applications such as hyperthermia and heat triggered drug delivery, surfactant coated nanoparticles are used; this study will be useful to design an experimental set-up and fine tune clinical applications.

## Conflicts of interest

There are no conflicts to declare.

## Supplementary Material
